# A Horticultural Therapy Program Focused on Succulent Cultivation for the Vocational Rehabilitation Training of Individuals with Intellectual Disabilities

**DOI:** 10.3390/ijerph17041303

**Published:** 2020-02-18

**Authors:** Yang Suk Joy, A-Young Lee, Sin-Ae Park

**Affiliations:** 1 Department of Horticultural Therapy, Graduate School of Agriculture and Animal Science, Konkuk University, Seoul 05029, Korea; flaxhurb2@naver.com; 2 Department of Environmental Health Science, Sanghuh College of Life Science, Konkuk University, Seoul 05029, Korea; danapre@nate.com; 3 Department of Bio and Healing Convergence, Graduate School of Konkuk University, Seoul 05029, Korea

**Keywords:** care farming, gardening, human issues in horticulture, intellectual disability, sociohorticulture

## Abstract

We aimed to develop a horticultural therapy program for the vocational rehabilitation of individuals with intellectual disabilities and examine its effects. Individuals with intellectual disabilities (*n* = 28, average age: 33.23 ± 4.9 years) were recruited from a welfare center in Jecheon, South Korea. They participated in eight weekly sessions of a horticultural therapy program consisting of common succulent cultivation techniques at a specialized succulent cultivation farm located in Jecheon, South Korea. Before and after the program, we assessed hand function (grip strength, pinch force, and hand dexterity, evaluated using a hand dynamometer, Jamar hydraulic pinch gauge, and grooved pegboard, respectively), emotional behavioral strategies (evaluated using the emotional behavioral checklist), and social skills (evaluated using the social skill rating system-teacher form). After participation in the horticultural therapy program, individuals with intellectual disabilities displayed significantly improved hand function, emotional behavior, and social skills (all *p* < 0.05). This study demonstrates the potential of horticultural therapy focused on succulent cultivation for the vocational training of individuals with intellectual disabilities. Future studies should investigate the effects of the program in a larger cohort.

## 1. Introduction

Employment forms an essential part of adult life for most individuals. As social beings, adult humans with and without disabilities aspire to work and serve their communities. Beyond its monetary value, meaningful work enhances not only one’s quality of life (physical and psychological well-being) but also their social independence [1,2]. For individuals with disabilities, work plays an essential role in their career development by providing valuable professional training [3], supplies an environment for socialization, and helps them define their roles in society. This increases self-image, eventually enabling individuals with disabilities to achieve higher levels of self-esteem [4].

Typically, the employment rate is much lower for individuals with disabilities. In South Korea, 36.9% of individuals with disabilities were employed, compared to 66.7% of those without disabilities [5]. The situation is even worse for individuals with intellectual disabilities, who have the lowest employment rate of all individuals with disabilities (21.8%) [6]. Most (66.7%) fail to acquire stable employment because their intellectual disabilities impair their ability to perform the required duties [5]. The World Health Organization defines the intellectually disabled as individuals that lack cognitive function, adjusting behaviors, and social skills [7]. These characteristics cause both intellectual and social limitations, making it difficult for individuals with intellectual disabilities to become and remain employed [8]. Furthermore, from a vocational perspective, individuals with disabilities face additional barriers: lack of self-confidence, work experience, job opportunities, and appropriate support for employment, as well as difficulties understanding workplace rules [9,10].

It is important to help find work for individuals with disabilities in their communities; however, it is even more important to offer social services that can connect these individuals with suitable work, considering their types of disability and individual capabilities [11,12]. Internationally, governments provide vocational rehabilitation services by implementing strategic policies so that individuals with disabilities can self-sustain throughout employment [13]. In South Korea, a law called Employment Promotion and Rehabilitation for the Disabled requires the government to develop and offer a variety of vocational rehabilitation opportunities for the disabled. These services include vocational skill development training, vocational adaptation training, protective employment, and employment intermediation. These services are provided by vocational training facilities for the disabled [13]. In this respect, primary industries, such as cultivating horticultural crops at farms and greenhouses, provide jobs that are good fits for individuals with intellectual disabilities, as they consist of relatively simple tasks for individuals without physical disabilities [14].

Succulents are plants with thickened, fleshy, and engorged parts that retain water to adapt to arid climates or soil environments [15]. Many home-growers and gardeners prefer succulents as houseplants due to their attractiveness and ease of care, and their cultivation and production have increased as the houseplant industry has expanded in recent years [16]. If properly planted, very little attention is required to successfully grow succulents. They are propagated mainly through stem or leaf cuttings, which is relatively easy and does not require sophisticated propagation techniques [17]. Individuals with intellectual disabilities have low intellectual capacities, but do not have limitations in physical functions and operations. For this reason, simple, somewhat monotonous and repetitive tasks of succulent cultivation that do not require specific skill sets can be a suitable job for individuals with intellectual disabilities looking for employment [14]. 

Horticultural therapy (HT) is defined as a complementary and alternative treatment in which a professional therapist uses horticultural activities to treat clients with special needs [18,19]. HT programs can serve as vocational rehabilitation for individuals with intellectual disabilities. Previous studies have reported HT programs for individuals with intellectual disabilities, examining changes in physically, psychologically, and socially related vocational functions [20,21,22]. One study administered a 22-session HT program to six female middle school students who have intellectual disabilities. In the program, the students conducted crafts using herbs and flowers and displayed improvements in hand function and emotional behavior [20]. Another study conducted an 18-session HT program for seven individuals with intellectual disabilities, in which the participants made topiaries at a living facility for people with disabilities. At the end of the program, the participants displayed increased vocational skills and adaptive behavior [21]. In a different study performed at another disabled living facility, 10 individuals with intellectual disabilities showed improved social skills and self-expression after a 33-session HT program [22]. However, these previous studies were all performed in a classroom in a school or welfare center as abridged versions of practical work-related skill training, not in a real work environment under the supervision of an employer. Community-based participation in a real work environment could deliver more efficient vocational training for individuals with disabilities [23]. 

Therefore, in this study, we developed an eight-session HT program focused on succulent plant cultivation at a real working farm for the vocational rehabilitation of individuals with intellectual disabilities and examined its effects. 

## 2. Materials and Methods

### 2.1. Participants 

Individuals with intellectual disabilities who were interested in a vocational rehabilitation training program based on horticultural activities were recruited for this study. Researchers visited a welfare center located in Jecheon, South Korea and provided information about the objective of the study, its requirements, and the program schedule and contents. Social workers at the welfare center posted and distributed a flier to advertise the program and to identify a recruitment pool for the study. The criteria for the recruitment were individuals (1) with an intellectual disability over 20 years of age, (2) with intention of landing a job through a vocational rehabilitation program, and (3) who were capable of using both hands and independent walking. The volunteers who declared the participation and their surrogates (guardians or social workers) signed in a written consent form before starting the study. Demographic information such as age, sex, and level of intellectual disability was obtained during an orientation session (Table 1). 

A total of 28 individuals with intellectual disabilities (16 males and 12 females) with an average age of 33.2 ± 4.9 years participated in this study. In South Korea, intellectual disability is classified into three levels based on IQ and social adaptability. Level 1 indicates an IQ < 34, level 2 an IQ of 35–49, and level 3 an IQ of 50–70 [24]. Level 1 and level 2 individuals with intellectual disabilities are classified as severely disabled. At level 1 disabilities, individuals are completely dependent on support from others to carry out daily activity. At level 2, individuals can perform simple tasks only in daily activities. These limitations work as barriers to guarantee employment and to successfully perform at work for individuals with intellectual disabilities at level 1 or 2 [24].

Among our participants, 11, 14, and 3 individuals had intellectual disability levels of 1, 2, and 3, respectively. The participants’ attendance in the program was 93.3%. This study was approved by the institutional review board of Konkuk University (7001355-201704-HR-171).

### 2.2. Development of an HT Program Focused on Succulent Cultivation

To develop an HT program focused on succulent cultivation for the vocational rehabilitation training of individuals with intellectual disabilities, we focused on the main activities required for succulent cultivation. To select these, 10 experts who run succulent farms in Jecheon, South Korea were interviewed either in person or by telephone. The experts were asked about the activities performed while cultivating succulents, their order, and their levels of difficulty. 

Based on this information, we designed an eight-session HT program with two levels (Table 2). We divided the program into basic and advanced courses based on the level of difficulty of the tasks. The former consisted of basic succulent propagation skills, such as making mixed soils, taking cuttings, and transplanting erect-type plants. The latter consisted of advanced skills for the management and maintenance of succulent plants, such as transplanting rosette-type plants, removing flower stalks, watering, and arranging plants into pots. Erect-type succulents, including most genera of the family Crassulaceae, have longer stems than those of rosette-type plants of the genus *Echeveria*, allowing easier handling. Therefore, erect- and rosetta-type succulents were used in the basic and advanced courses, respectively.

### 2.3. Implementation of the Eight-session HT Program for the Vocational Rehabilitation Training of Individuals with Intellectual Disabilities

The eight-session HT program was conducted between May and November 2018 on a professional succulent cultivation farm located in Jecheon, South Korea. The weekly sessions were approximately 120 minutes long. The HT program was run by five horticultural therapists certified by the Korean Horticultural Therapy Association, with the assistance of two social workers from the community welfare center for the disabled.

Each session contained three steps: introduction, development, and closing (Table 3). In the introduction step, the horticultural therapists conducted work preparation and safety education. In the development step, they introduced and demonstrated horticultural activities to the participants, and then the participants practiced these activities. For better vocational rehabilitation training, increased understanding and learning skills are required for individuals with intellectual disabilities to cultivate succulents. Therefore, we sought to increase the effectiveness of training as follows: 1) the horticultural therapists provided one-on-one training to individuals with consideration of each participant’s degree of disability; 2) the participants were asked to imitate other participants’ work postures, to learn skills through cooperative activity; and 3) the participants were asked to repeat and refine the horticultural work motions during the session. In the closing step, the horticultural therapists asked the participants to tidy up after the horticultural activity.

In the first session, the horticultural therapists taught the participants how to make mixed soils and how to distinguish different types of artificial growth media for growing plants, such as perlite, peat moss, coarse sand, and coco-peat, by touching and smelling. The horticultural therapists also explained the ratios of these growth media in mixed soils and how to blend a growing medium for further horticultural activities. Following the instructions, the participants mixed the soil, ensured uniform soil particle size, and added water to the growing medium.

In the second session, the horticultural therapists taught various leaf-cutting techniques. Following the instructions, the participants classified the samples by leaf size and succulent type. They filled trays with the mixed soils and placed the leaf cuttings on the propagating trays using their bare hands. 

In the third session, the horticultural therapists taught various stem-cutting techniques using scissors. Erect-type succulents, such as *Sedeveria Letizia and Sedum* ‘Alice Evans’, were used in the session. The participants filled in 50 pots with mixed soils and added stem cuttings made using scissors. These cutting practices were repeated until the participants were comfortable with the skills learned in the sessions. 

In the fourth session, the horticultural therapists taught the participants how to transplant plants using erect-type succulent plants, such as *Crassula ovata* and *Sedum corynephyllum*, which have high stems that make grasping them relatively easy for the intellectually disabled. The participants arranged cotyledons and roots and transplanted the plants into 8-cm plastic pots. 

In the fifth session, the first of the advanced sessions, the horticultural therapists taught the participants how to transplant plants using rosette-type succulent plants, such as *Echeveria* ‘Puli-lindsayana’ and *Echeveria* ‘Esther’ *f. cristata.* These rosette-type succulents do not have stems between the roots and leaves, making grasping relatively difficult for the intellectually disabled and requiring more detailed skills. These transplanting sessions were repeated 20 times to familiarize the participants with the tasks involved. 

In the sixth session, the horticultural therapists taught the participants how to remove flower stalks, including how to distinguish flower stalks from stems and how to cut them off with scissors. The participants conducted these tasks using succulents with high flower stalks, such as *Echeveria* ‘Minibelle’ and *Echeveria* "Pretty in Pink." 

In the seventh session, the horticultural therapists taught the participants how to water the plants and how to choose an appropriate irrigation method, time, and amount of water according to the type of succulent. The participants practiced various watering activities: distinguishing plants requiring irrigation, bottom-watering plants, and watering with a hose or watering can. 

In the eighth session, the horticultural therapists taught the participants how to arrange plants in a pot. The participants chose and transplanted a total of five succulents, considering the characteristics of the plants. They used cultivation skills learned throughout the HT program to complete the tasks in this step.

### 2.4. Vocational Function Assessments

We worked collaboratively with the social workers to conduct vocational function assessments before and after the eight-session HT program, assessing physical, psychological, and social functions.

#### 2.4.1. Physical Functions

A series of hand function tests was conducted to assess the physical function of the participants. Grip strength, pinch force, and hand dexterity were measured using a Jamar hydraulic hand dynamometer (5030J1; Sammons Preston, Bolingbrook, IL, USA), a Jamar hydraulic pinch gauge (749805; Sammons Preston), and a grooved pegboard (32025; Lafayette Instrument Co., Lafayette, IN, USA), respectively. For grip strength and grip force, we used the highest values attained (in kg) in three test repetitions. Hand dexterity was evaluated using the time the participants needed to perform a given task. All measurements were evaluated using the dominant hand of each participant.

#### 2.4.2. Psychological Functions 

Assessments of emotional behavior strategies were conducted by observing activities during HT. To evaluate emotional behavior strategies, the emotional behavioral checklist (EBC) was used, which is a part of the McCarron–Dial evaluation system that assesses the vocational competence of people with disabilities [25]. The checklist consists of 35 questions, and a lower score indicated more positive emotional behavior. The three-point Likert scale consisted of seven subcategories, including impulsivity-frustration, anxiety, depression-shrinking, socialization, aggression, derealization, and self-concept. The Cronbach’s α coefficient of the survey was 0.94.

#### 2.4.3. Social Functions

Social skills were assessed using the social skill rating system-teacher form (SSRS-TF) [26,27]. The assessment included 30 questions about various behaviors related to interpersonal relationships that individuals with intellectual disabilities are required to adjust in their society or community. The responses were scored on a three-point Likert scale, in which a higher score indicated a higher degree of social skills. Cronbach’s α coefficient of the survey was 0.96. Assessments of social skills were also conducted by observing activities in the HT program.

### 2.5. Satisfaction Survey

A previously described satisfaction survey was tailored to the HT program [28] and was completed by the participants after the eight-session HT program. This survey was composed of seven questions, addressing the participants’ overall satisfaction with the HT and the duration of the HT and its sessions, their preferences for activities performed in HT, their wish to continue participating in the HT, and their intent to recommend the HT to other individuals with intellectual disabilities.

### 2.6. Data Analysis

To analyze differences of the participants’ test scores before and after the HT program, paired t-tests were conducted in SPSS (version 25.0; IBM Corp., Armonk, NY, USA). Values of *p* < 0.05 were considered statistically significant. Cronbach’s α coefficient was obtained by reliability analysis. Demographic information and satisfaction with the HT program were analyzed using MS Excel software (Microsoft Office 2007; Microsoft Corp., Redmond, WA, USA).

## 3. Results

### 3.1. Physical Functions

Upon completion of the eight-session program, participants exhibited significant improvements in grip strength, pinch force, and hand dexterity (Table 4).

### 3.2. Psychological Functions

Emotional behavior strategies were significantly improved, as evidenced by a significant decrease in mean EBC score (Table 5). Scores for subcategories, such as impulsivity-frustration, depression-shrinking, socialization, and self-concept, were also significantly decreased [25].

### 3.3. Social Functions

The participants experienced increases in their SSRS-TF scores (Table 5). People with higher social skills are more likely to obtain employment and remain employed and score higher on measures of integration [27].

### 3.4. HT Program Satisfaction

The participants reported that they were ‘‘very satisfied’’ (46.4%), ‘‘satisfied’’ (39.3%), ‘‘normal’’ (10.3%), and ‘‘not satisfied’’ (3.6%) with the HT program. In addition, 46.4% of the participants were satisfied with the 120-min activity time per session. The 53.6% of participants who reported feeling ‘‘not satisfied’’ felt that 60 or 90 min sessions would have been adequate. All participants were either ‘‘very satisfied’’ (57.1%) or ‘‘satisfied’’ (42.9%) with the weekly session frequency. The most preferred HT activities were ‘‘leaf-cutting’’ (22.8%), ‘‘mixing soil’’ (21.3%), and ‘‘stem-cutting’’(18.0%). In addition, 92.9% of the participants reported that they wished to continue participating in the HT program, and 96.4% of the participants would recommend it to other individuals with intellectual disabilities.

## 4. Discussion

The HT program as vocational training presented in this study was effective in improving hand function, emotional behavior strategies, and social skills in individuals with intellectual disabilities. These effects were likely derived from the physical, psychological, and social aspects of the HT activities, as well as from their operation on a working farm. 

The improved hand function was likely due to the repetition of simple hand motions while cultivating succulents. Many horticultural activities comprise repeated reaching-grasping motions that require fine motor skills [29]. Repeating simple motions helps with learning motor skills, allowing the participants to master skills and gain agility [30,31]. The repetition of simple motions could also affect neuroplasticity, fluidly changing the function and structure of the brain [32]. For the participants, the brain function triggered by repetitive horticulture activities led to the effective learning of succulent cultivation skills. The succulent plants used in this program have fast growth and propagation characteristics, which would be ideal for coordinating repetitive horticultural activities like cutting, potting, and transplanting [16,17].

The improved grasp force was likely due to the different tasks included in the HT program [29,30], including mixing soils, grasping and filling pots, and watering with a hose or a watering can, where the participants were forced to practice ball grasping and cylindrical grasping. Grasping actions require hand force, which can be measured by a hand-dynamometer using a similar motion [29,30]. Similarly, the pinch force of the participants was improved, likely because of the frequent use of pinching motions during the horticultural activities. Grasping plants and trays and transplanting cuttings involve lateral prehension, fingertip prehension, and palmar prehension, all of which require two- or three- finger force that can be measured by a pinch gauge using a similar motion [29,30]. The improvements in grasp and pinch force account for the improved hand dexterity observed in this study. Detailed and complex motions require development of sensory-motor components, such as grasp force, pinch force, and stereognosis to ensure coordination between the eyes and the fine motor skills of the hand [33,34]. In this sense, these improvements in hand functions could provide the intellectually disabled with a foundation to develop further detailed hand skillsets for more sophisticated tasks. The hand muscles are used most in horticultural activities, including the thenar eminence and hypothenar eminence [35].

A previous study also reported improved physical function in individuals with intellectual disabilities after an HT program [36,37,38]. Ten individuals with intellectual disabilities participated in a 38-session HT program involving planting, flower arrangement, and crafting with plants, and displayed significant improvements in grasp force and hand tapping [36]. In another study, four high school students with intellectual disabilities repeated flower arrangement tasks in a 20-session HT program. At the end of the study, the participants displayed increased hand dexterity [37]. 

Physical function is a critical aspect of vocational rehabilitation for individuals with intellectual disabilities. Specifically, hand function is essential for the duties required for many jobs [39]. The majority of people with intellectual disabilities have limitations in fine motor skills rather than gross motor skills, considering their relatively normal physical development [40]. For this reason, for vocational rehabilitation, improvement of hand skills is crucial. The fine motor skills gained from vocational training help individuals with intellectual disabilities to search for and acquire various jobs [39]. Given its focus on hand dexterity, horticultural activity is relatively suitable vocational training for people with intellectual disabilities.

The improvements in emotional behavior strategies are likely derived from the fact that the HT activities were designed to be easily performed by the participants, considering the difficulties of these tasks for the disabled. The horticultural activities included in the vocational rehabilitation program in the present study were repetitive, easy tasks, allowing the participants to experience the success of achieving goals at work. Particularly, succulent plants, compared with other types of plants, have higher propagation rates and are easy to cultivate and manage. For this reason, the risk of failure in performing horticultural activities is remarkably low with succulents, which provides opportunities for achievement [16,17].

Individuals with intellectual disabilities tend to display low motivation or lethargy, which is attributable to many frustrations or rejections in their past lifetime experiences [41]. Ensuring successful experiences by designing work with proper difficulty levels provides a sense of accomplishment and increases personal motivation, which together positively affect emotional behavior [42].

Generally, people are easily bored by the monotony of performing repeated, simple tasks, decreasing morale for the work [43]. However, horticultural activities that involve the use of living plants are goal-driven and require continual maintenance [44,45]. These characteristics help individuals remain interested and motivated to complete the work. 

Furthermore, working with plants has demonstrated psychophysiological effects, invoking positive emotional changes. For instance, participants who participated in activities using plants showed increased alpha-waves in the brain, stabilized brain blood flow, and heart beats [46,47]. In another study, 30 adults with intellectual disabilities who participated in horticultural activities exhibited steadiness of the autonomic nervous system and reductions in the stress hormone, cortisol [48]. In addition, horticultural activities increase serotonin, a depression-related hormone, resulting in improved cognitive function [49]. These emotional and psychological benefits of horticultural activities could have positively affected the emotional behavior of the participants in the present study. 

Previous studies have also reported the positive effects of HT programs on the emotional behavior strategies and psychological behavior of individuals with intellectual disabilities [36,50,51,52]. For example, after participating in a 22-session HT program on hydroponic lettuce cultivation, 14 individuals with intellectual disabilities displayed significant improvements in emotional behavior and related subcategories, such as impulsivity-frustration, socialization, and aggression [50].

The development of social skills observed in this study can be attributed to the participants’ cooperation with their colleagues in a real work environment. Individuals with intellectual disabilities often develop poor social skills and an inability to form healthy relationships [53]. Through HT programs, these individuals can experience social interactions and build relationships by working as a team in a natural manner to perform activities [54]. In addition, our HT program provided repeated opportunities to practice many social skills (greetings, arrangements, assignments, etc.), which may have improved the social skills of the participants.

The social rehabilitation effects of HT programs are important, allowing the opportunity for recovery to individuals who are marginalized within society [55]. Most HT programs are operated in groups rather than individually. Therefore, through these activities, individuals with intellectual disabilities gain realistic opportunities for social practice with colleagues, experiencing positive social experiences rather than isolation [56]. As our program was conducted on a working succulent farm, the participants had a chance to develop vocational skills and learn meaningful skill sets that can be readily applied to actual occupations, while interacting with colleagues who shared the same tasks and responsibilities [57]. In this way, the individuals with intellectual disabilities were able to develop and hone practical social skills applicable to gaining and maintaining employment. 

Previous studies reported similar improvements in the social skills of people with intellectual disabilities after HT programs [22,52,58,59]. Lee observed significantly improved social skills and self-expression in ten people with intellectual disabilities during a 33-session HT program in which they participated in indoor horticultural activities such as planting, creating topiaries and floral arrangements, and crafting using plants [22]. In another study, 24 women with intellectual disabilities who completed a 24-session HT program involving indoor horticultural activities had significantly better social and interpersonal relationship skills compared to a control group [58].

Individuals with intellectual disabilities suffer the full range of psychological health problems; as a result, they are more vulnerable to behavioral disorders, resulting in employment difficulties [60]. The significantly positive psychological and functional effects observed in this study demonstrate the importance of vocational rehabilitation efforts. In addition to improved psychological functioning, improved social functioning can ensure stable employment and improve the adaptability of those with intellectual disabilities in society [61]. Psychological functions, such as confidence and motivation for work, and social interactions, such as small talk in the workplace, are important individual factors for the successful employment of individuals with intellectual disabilities [62,63]. As these factors can be simultaneously developed and practiced in HT programs, these programs can offer successful vocational rehabilitation to individuals with intellectual disabilities.

## 5. Conclusions

Our HT program displayed positive vocational rehabilitation effects for individuals with intellectual disabilities. This study demonstrates the potential of HT programs as vocational training that could be used to develop employment skills based on the characteristics of an individual’s disabilities. To confirm the effects of the present study, future experiments including a control group will be required. In addition, efforts should be made to customize or improve the design of HT programs to consider various disabilities and occupations of different difficulty levels. For example, the development and assessment of HT programs with diverse horticultural crops (e.g., fruit trees, ornamentals, vegetables, and mushrooms) would provide enhanced job training for people with intellectual disabilities.

## Figures and Tables

**Table 1:** Demographic characteristics of participants [% (*N*)].

Variable	Horticultural Therapy Group (*n* = 28)
Gender	
Male	57.1 (16)
Female	42.9 (12)
Age	
20−30	20.0 (3)
30−40	53.3 (8)
Over 40	26.7 (4)
Disability rating ^1^	
Level 1	39.3 (11)
Level 2	50.0 (14)
Level 3	10.7 (3)

^1^ Level 1: IQ < 34; level 2, IQ 35–49; level 3, IQ 50–70 [23].

**Table 2:** Sessions in the eight-session horticultural therapy (HT) program.

Stage	Session	Horticultural Activity
Basic	1	Making mixed soils
	2	Leaf-cutting
	3	Stem-cutting
	4	Transplanting plants
Intensive	5	Transplanting plants
	6	Removing flower stalk
	7	Watering
	8	Arranging plants into a pot

**Table 3:** Outline of the HT program sessions.

Step (Time)	Contents	
Introduction (10 min)	Preparing work	Greeting and checking in
		Attendance checking
		Putting on name tag and wearing groves
	Safety education	Explaining safety rules and precautions in the farm
		Introducing work tools and agricultural facility used in each session
Development (100 min)	Introducing horticultural activity	Demonstration of horticultural activity step by step for participants
	Practicing cultivation succulents Ⅰ	One-on-one training to individuals
	Resting	Washing hands and drinking water
	Practicing cultivation succulents Ⅱ	Cooperation group activity
Closing (10 min)	Announcing next session	-
	Wrapping and tidying up	Cleaning work place
		Taking off name tag and groves
		Closing

**Table 4:** Effects of the HT program on the physical functions of individuals with intellectual disabilities (Mean ± SD).

Variable	Pretest	Post-test	*p* ^1^
Grip strength (kg)	14.55 ± 5.75	17.29 ± 6.32	0.000 ***
Pinch force (kg)	11.48 ± 3.81	14.40 ± 5.13	0.000 ***
Fine motor skill (s)	263.93 ± 174.02	230.14 ± 145.34	0.005 **

^1^ *** and ** indicate significant differences at *p* < 0.001 and *p* < 0.01, respectively.

**Table 5:** Effects of the HT program on the psychological and social functions of individuals with intellectual disabilities (Mean ± SD).

Variable	Pretest	Post-test	*p* ^3^
**Psychological behaviour ^1^ (score)**	22.96 ± 12.15	16.21 ± 13.72	0.007 **
Impulsivity-frustration	4.03 ± 2.68	2.96 ± 2.31	0.033 *
Anxiety	3.32 ± 2.84	2.50 ± 2.04	0.115
Depression- shrinking	3.53 ± 2.36	2.00 ± 2.21	0.013 *
Socialization	3.46 ± 2.88	2.17 ± 2.32	0.042 *
Aggression	2.07 ± 2.52	1.71 ± 3.06	0.609
Self-concept	3.61 ± 2.10	2.64 ± 2.56	0.011 *
Derealization	2.92 ± 2.41	2.21 ± 2.20	0.081
Social skill ^2^ (score)	25.53 ± 13.08	43.35 ± 11.73	0.000 ***

^1^ Derived using the emotional behavioral checklist (EBC), which is a part of the McCarron–Dial evaluation system. A lower score indicates more positive emotional behavior [25]. ^2^ Derived using the social rating system-teacher form (SSRS-TF). A higher score indicates a higher degree of social skills [26,27]. ^3^ ***, **, and * indicate significant differences at *p* < 0.001, *p* < 0.01, and *p* < 0.05, respectively.
